# TargetMine 2022: a new vision into drug target analysis

**DOI:** 10.1093/bioinformatics/btac507

**Published:** 2022-07-27

**Authors:** Yi-An Chen, Rodolfo S Allendes Osorio, Kenji Mizuguchi

**Affiliations:** Artificial Intelligence Center for Health and Biomedical Research (ArCHER), National Institutes of Biomedical Innovation, Health and Nutrition, Osaka 567-0085, Japan; Artificial Intelligence Center for Health and Biomedical Research (ArCHER), National Institutes of Biomedical Innovation, Health and Nutrition, Osaka 567-0085, Japan; Artificial Intelligence Center for Health and Biomedical Research (ArCHER), National Institutes of Biomedical Innovation, Health and Nutrition, Osaka 567-0085, Japan; Institute for Protein Research, Osaka University, Osaka 565-0871, Japan

## Abstract

**Summary:**

We introduce the newest version of TargetMine, which includes the addition of new visualization options; integration of previously disaggregated functionality; and the migration of the front-end to the newly available Bluegenes service.

**Availability and Implementation:**

TargeteMine is accessible online at https://targetmine.mizuguchilab.org/bluegenes. Users do not need to register to use the software. Source code for the different components listed in the article is available from TargetMine’s organizational account at http://github.com/targetmine.

**Supplementary information:**

Supplementary data are available at *Bioinformatics* online.

## 1 Introduction

The last decade has seen a steady increase in the number of studies related to multi-omics analysis (Krassowski *et al.*, 2020; Tarazona *et al.*, 2021). References for ‘Multi-omics Analysis’ reviews listed on PubMed increases from 4 in 2012 to 145 in 2021 (https://pubmed.ncbi.nlm.nih.gov. Last accessed, 1 February 2022). Multi-omics analysis can not only be used to improve the classification of biological data but also for the prediction of variables (such as clinical outcomes), and it might even have the potential to elucidate regulatory mechanisms that include several molecular layers (Tarazona *et al.*, 2021).

A main challenge in multi-omics analysis lies in data integration (Canzler *et al.*, 2020; Krassowski *et al.*, 2020; Tarazona *et al.*, 2021). Approaches on data integration include early integration—data are concatenated into a single matrix; intermediate integration—jointly analyze different omics layers together; and late integration—integrate the analysis results (Adossa *et al.*, 2021). This categorization has been extended by Picard *et al.* (2021) to also consider mixed and hierarchical integration strategies. At the same time, the development of platforms for the storage of multi-omics data also remains a strong research focus, with (Eloe-Fadrosh *et al.*, 2021; Tang *et al.*, 2022; Zhou *et al.*, 2022) being only a few examples across different domains, all of them reported in this year’s Nucleic Acid Research’s special issue on Databases (Rigden and Fernández, 2022).

In this context, the TargetMine Data Warehouse has evolved into an integrative data analysis platform. TargetMine incorporates various types of omics data, sourced from a variety of data sources and models to provide a deep coverage of the biological data space, with a focus on target prioritization and broad-based biological knowledge discovery (Chen *et al.*, 2011, 2019). Consolidated as a useful resource for the drug discovery scientific community (as suggested by the number of citations of the original paper as recorded by PubMed), through the integration of new data from different, heterogeneous sources, and by providing new widgets for its analysis, TargetMine continues to strive in becoming an integral solution to multi-omics data analysis, especially in terms of data storage and biological interpretation (Tarazona *et al.*, 2021).

## 2 New in TargetMine

### 2.1 Integration and new visualization tools

Up until now, TargetMine also included an *Auxiliary Toolkit* (Chen *et al.*, 2016), accessible through a separated user interface. This has now been integrated into a single-user experience. The display of a *Composite Network Graph*, added to report pages of gene lists, allows interactive visualization of gene-to-gene interactions among the list members, together with their relation to other genes, microRNA, chemical compounds and/or transcription factors found within TargetMine. Similarly, the *Enrichment Display Graph*, also included in the gene list report page, shows through bar graphs and heatmaps, the proportion of genes with a given annotation compared with the annotation of the whole genome, or how the individual genes in the list are matched to the corresponding enriched elements, respectively.

**Fig. 1.**
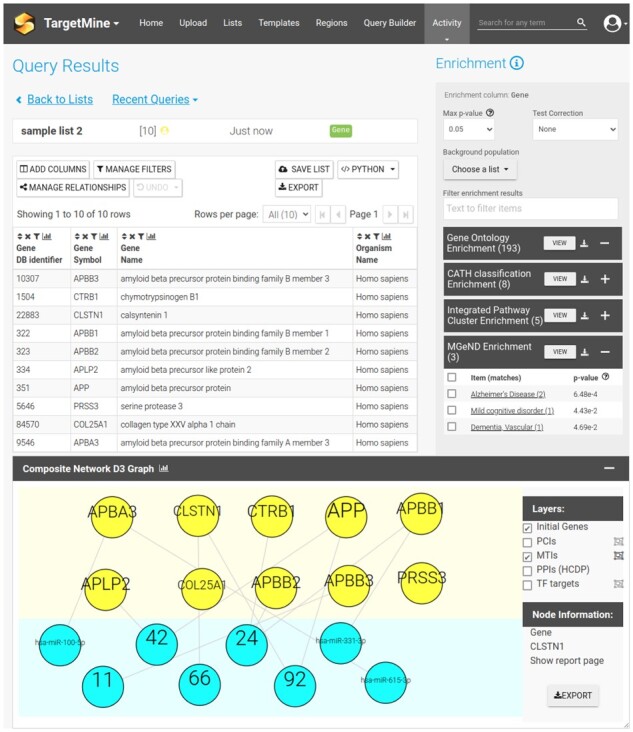
Sample image of TargetMine’s new interface for a list of genes. Elements have been slightly adjusted for better display here. MicroRNA associations to original genes are shown in graph format. New MGeND enrichment for the list of genes is also shown

Completely new display widgets have also been added to TargetMine. The gene report page now includes a *Gene Expression Graph* (see Fig. 1); and the report page for chemical compounds has now a *Bio-activity Graph*. As suggested by their names, both these graphs allow to dynamically inspect either the expression or bio-activity levels of individual genes or chemical compounds, respectively. Whilst the first includes controls to handle the display at different levels of detail where the gene expression is measured; the second provides controls to clearly identify different assays.

Details and user guides for all the aforementioned visualization tools are provided as Supplementary Material to this article.

### 2.2 Bluegenes migration

TargetMine is based on InterMine (Kalderimis *et al.*, 2014; Smith *et al.*, 2012), a data warehousing system that provides easy query and analysis of various heterogeneous data sources. Paired to InterMine, a new front-end named Bluegenes (https://github.com/intermine/bluegenes), meant to replace the old Java Server Pages (JSP)-based interface has been released.

As several customly implemented elements of TargetMine were implemented as components of the JSP-based interface, they all needed to be refactored into new *Bluegenes tools*. Figure 1 shows an example of the new interface used for TargetMine, in particular, the one used to report information of a list of genes. Users familiar with the application will notice the new, modern feel and look achieved with the new front-end.

One major advantage of this approach is that each element can be implemented as its own *project*, and thus can be individually maintained (i.e. is kept on its own GitHub repository). An extensive list of all the migrated tools and their corresponding repositories is provided as Supplementary Material.

### 2.3 New data sources

In order to continuously improve the coverage of the biological data space, some new data types and sources were added. These new data sources include protein binding pockets from PoSSuM (Ito *et al.*, 2015), genomic variant with clinical annotation from MGeND (see Fig. 1) (Kamada *et al.*, 2019), clinical trial data from WHO (https://trialsearch.who.int/. Last accessed, 11 March 2022) and also genome annotations from NCBI (https://www.ncbi.nlm.nih.gov/genome. Last accessed, 11 March 2022). New data are accommodated by extending the data model currently used by TargetMine, which can be generally described as an Object Oriented definition, transpilled into a Relational database for storage purposes. More details on how this is implemented can be found in (Chen *et al.*, 2011). Applications of the new additions will be reported elsewhere.

## 3 Discussion

We believe TargetMine to be a highly valued data warehouse within the drug discovery research community, as proved by the continuous access that it has on a daily basis, from countries across five continents. As a response to the support shown by the community, we constantly strive to improve the service, with monthly data updates and constant software updates being a proof of our commitment toward this end.

Here, we introduced some of the major updates made to TargetMine over the past couple of years, namely, its migration to a new front-end and the development of new visualization widgets, customly targeted to specific data elements within the repository.

## Supplementary Material

btac507_Supplementary_Data

## Data Availability

The source code for TargetMine is available under an MIT License at http://github.com/targetmine/targetmine-gradle. Source code for different the components listed in the manuscript are available from the following repositories under TargetMine’s organizational GitHub account: bluegenes-tool-compositenetworkd3-graph, bluegenes-list-enrichment-visualizer, bluegenes-tool-gene expression-graph and bluegenes-tool-bioactivity-graph.
