# Supplementary material for: TargetMine 2022: a new vision into drug target analysis
Source: Bioinformatics. 2022 Jul 27;38(18):4454–6. doi: 10.1093/bioinformatics/btac507 (PMC9477527; doi:10.1093/bioinformatics/btac507)
Supplement: btac507_Supplementary_Data [file btac507_supplementary_data.pdf]

TargetMine 2022: A new vision into drug target analysis

Supplementary Information

The following document is a supplementary documentation to the homonymously titled draft submission recording the TargetMine development over the past few years.

This document is divided into the following sections: 1. *Quick Start* gives a fast introduction to TargetMine following the example of using a simple search for a specific protein ; 2. *Integration and new Visualization Tools* provides a user guide style introduction to the new features found in TargetMine; finally 3. *Bluegenes Migration* gives details on all the customly developed components previously developed for TargetMine that have been migrated to the new Bluegenes style.

1. Quick Start

The following example introduces the *search* functionality of TargetMine. Searching and being able to inspect search results is probably the simplest way to use TargetMine, to be performed by probably every user of the tool, and thus we believe it to be a perfect introduction to our work.

The *search* functionality is available immediately at the front page of our application, as shown by the highlighted red squares in Fig. 1.

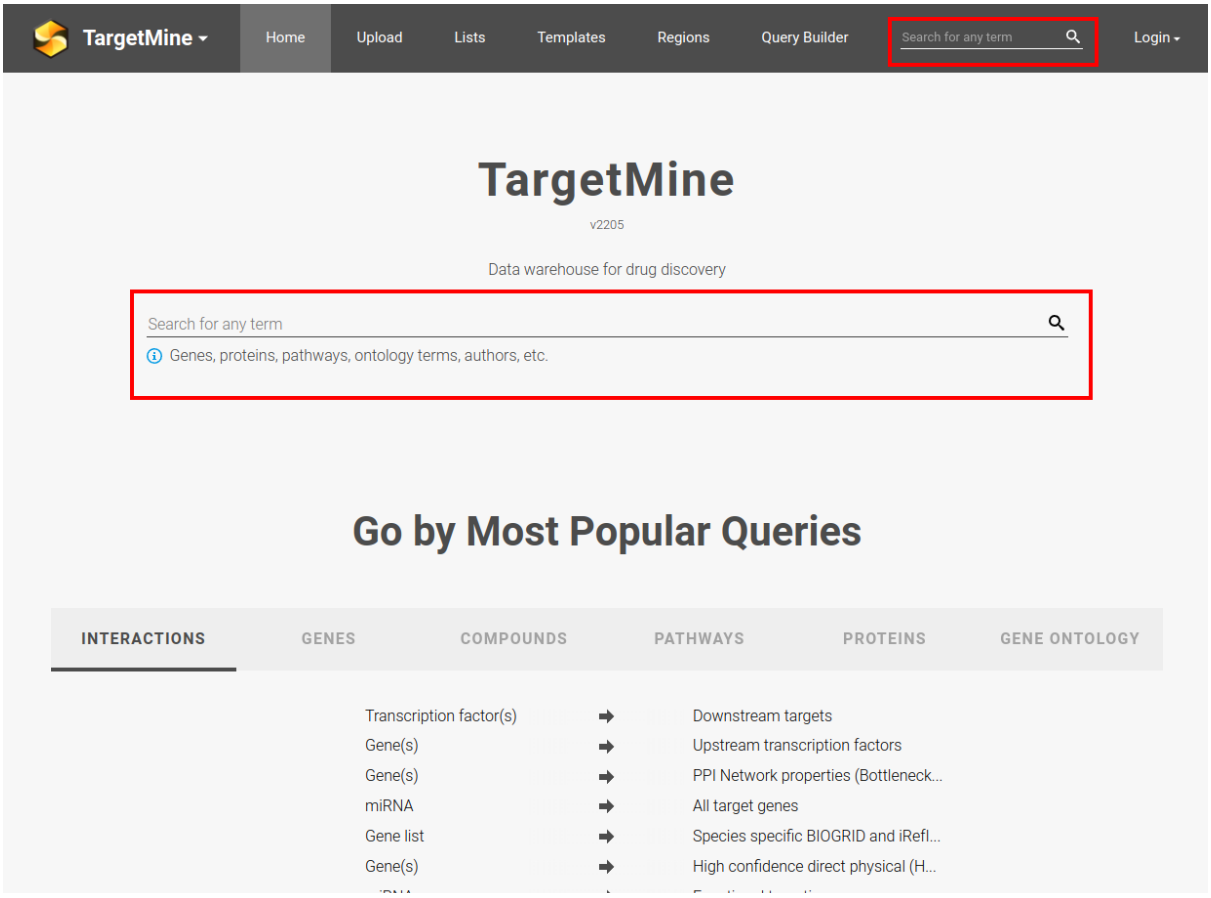

Figure 1. Search boxes available in TargeMine's starting page. Notice that the top-right side box remains available throughout the use of the tool.

As text is entered in the corresponding box, a list of suggested results is dynamically loaded and presented to the user for selection (as shown on the left side of Fig. 2). For the purpose of this example, we input the identifier “APP” and click on “Show all results”. Upon selection, the list of results is as the one shown on the right side of Fig. 2.

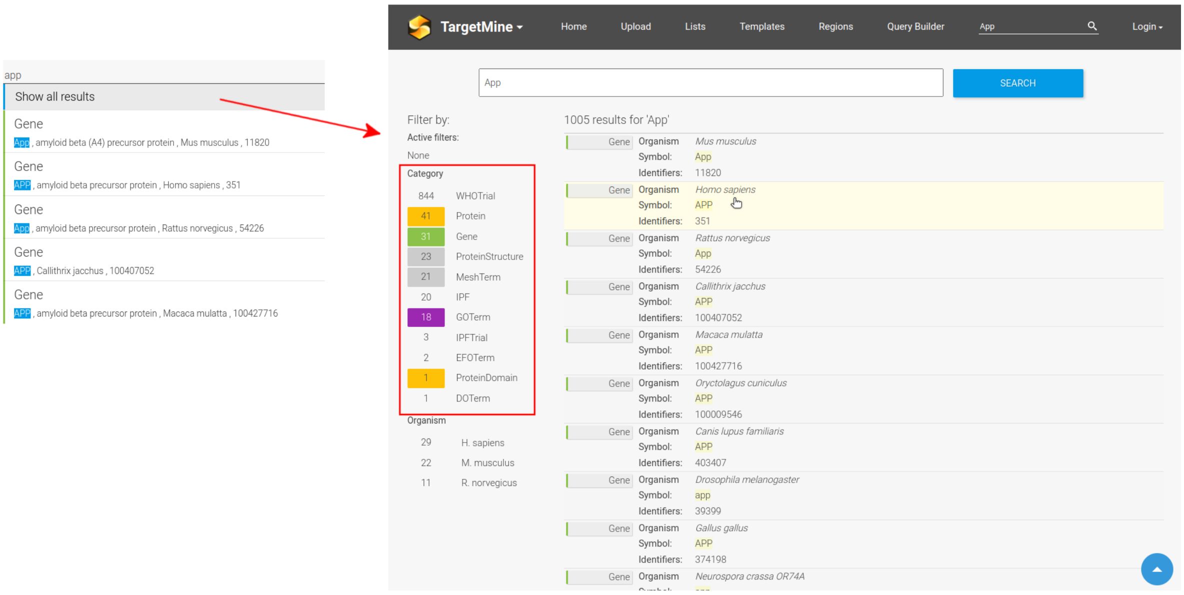

Figure 2. Searching for the term “app” on TargetMine and the corresponding list of results. The variety of results are shown as different categories.

Notice that multiple categories of results are potentially available (see highlighted box in Fig. 2). By clicking on any single category, only the corresponding results will be shown. By choosing a category, multiple selection of elements also becomes available. For the purpose of this example, we will click on a single element rather than selecting a list of elements, as shown in the highlighted result of Fig. 2.

On selection of an individual element, the corresponding report page is shown (see Fig. 3). The information listed on the report page comes from the integration of the different sources that make up the core of TargetMine. In our example, it is possible to find, for example, information related to the gene’s homology, disease associations and related pathways, among others.

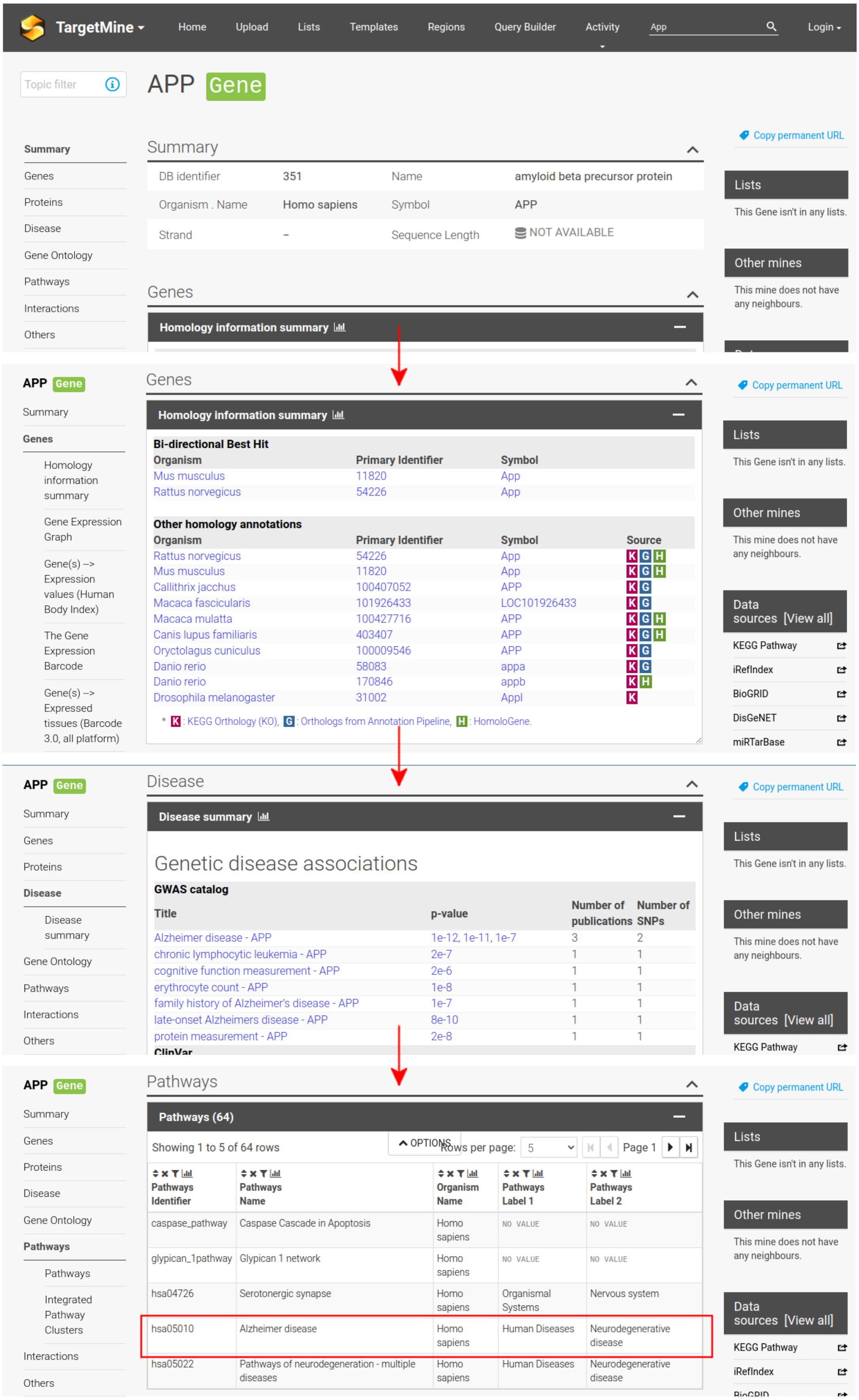

Figure 3. Report page displayed for Gene element APP. Notice how different content is available as we scroll down the page.

It is also possible to navigate through the data contained in TargetMine using the displayed associations between the current element and those of other categories within the system. In our example, by clicking on “hsa0510”, the identifier for KEEG’s Alzheimer disease pathway (see the highlighted box of Fig. 3), it is possible to access its corresponding report page, together with a list of all 384 genes that (at the time of writing) are part of it (as shown in Fig. 4).

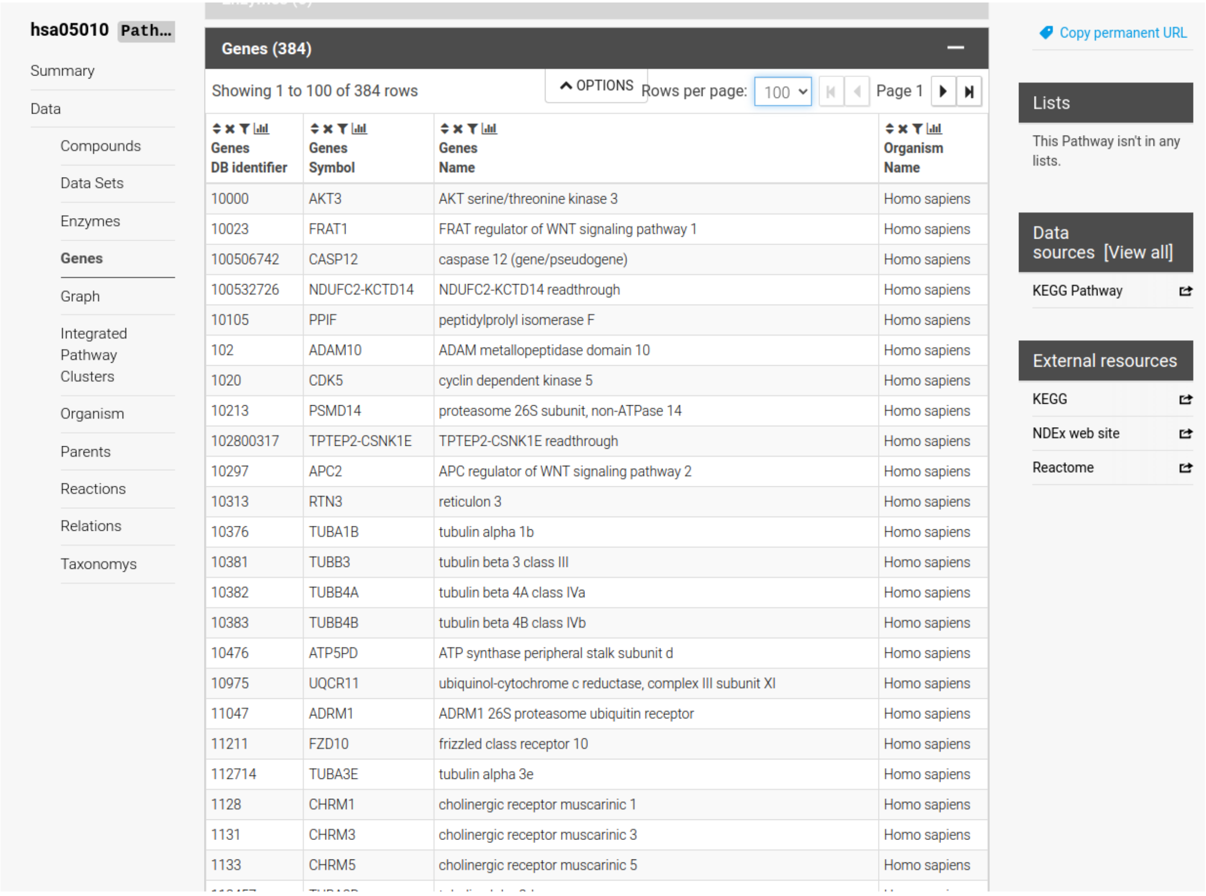

Genes DB identifier	Genes Symbol	Genes Name	Organism Name
10000	AKT3	AKT serine/threonine kinase 3	Homo sapiens
10023	FRAT1	FRAT regulator of WNT signaling pathway 1	Homo sapiens
100506742	CASP12	caspase 12 (gene/pseudogene)	Homo sapiens
100532726	NDUFC2-KCTD14	NDUFC2-KCTD14 readthrough	Homo sapiens
10105	PPIF	peptidylprolyl isomerase F	Homo sapiens
102	ADAM10	ADAM metallopeptidase domain 10	Homo sapiens
1020	CDK5	cyclin dependent kinase 5	Homo sapiens
10213	PSMD14	proteasome 26S subunit, non-ATPase 14	Homo sapiens
102800317	TPTEP2-CSNK1E	TPTEP2-CSNK1E readthrough	Homo sapiens
10297	APC2	APC regulator of WNT signaling pathway 2	Homo sapiens
10313	RTN3	reticulon 3	Homo sapiens
10376	TUBA1B	tubulin alpha 1b	Homo sapiens
10381	TUBB3	tubulin beta 3 class III	Homo sapiens
10382	TUBB4A	tubulin beta 4A class IVa	Homo sapiens
10383	TUBB4B	tubulin beta 4B class IVb	Homo sapiens
10476	ATP5PD	ATP synthase peripheral stalk subunit d	Homo sapiens
10975	UQCR11	ubiquinol-cytochrome c reductase, complex III subunit XI	Homo sapiens
11047	ADRM1	ADRM1 26S proteasome ubiquitin receptor	Homo sapiens
11211	FZD10	frizzled class receptor 10	Homo sapiens
112714	TUBA3E	tubulin alpha 3e	Homo sapiens
1128	CHRM1	cholinergic receptor muscarinic 1	Homo sapiens
1131	CHRM3	cholinergic receptor muscarinic 3	Homo sapiens
1133	CHRM5	cholinergic receptor muscarinic 5	Homo sapiens

Figure 4. The list of genes that are part of Pathway hsa05010, as shown in the pathway’s report page of TargetMine .

2. Integration and new Visualization Tools

a. Composite Network graph

The *Composite Network graph* has been included in the report page used for lists of genes. The graph is made up from five distinctive layers: the initial genes in the list; a list of linked chemical compounds (PCIs); the list of miRNA targets (MTIs) that interact with the initial genes; a list of genes that interact with the ones in the initial list (PPIs - HCDP); and finally, a list of transcription factors. An example of a Composite network file is shown in Fig 5.

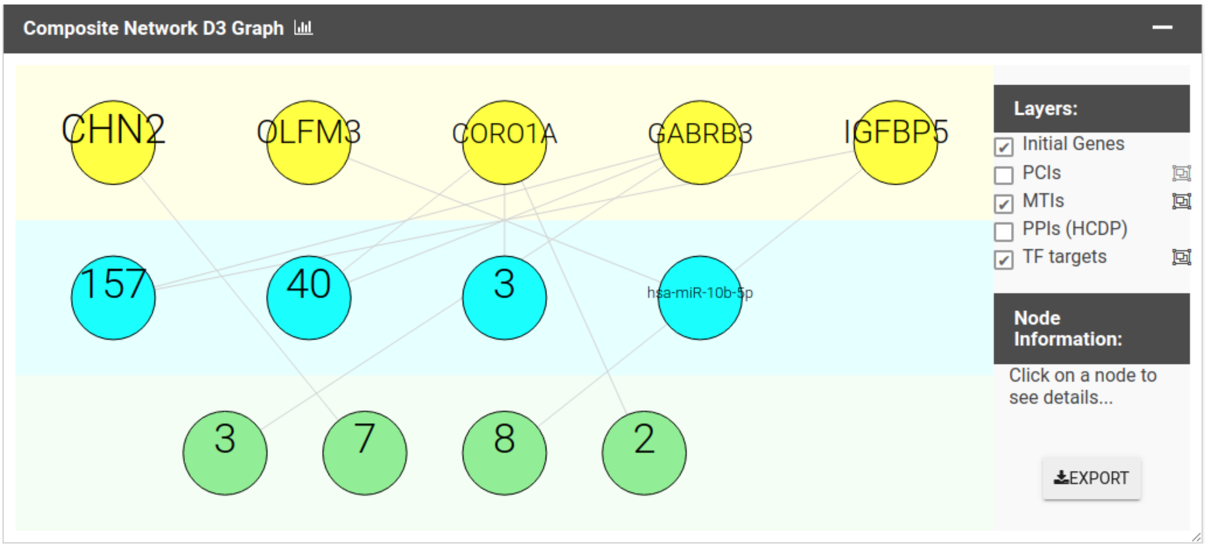

Figure 5. Composite Network graph.

Notice that, for the graph depicted on Fig. 5, only the initial genes, and their interactions to miRNA and transcription factors are shown.

The user can reposition nodes (within their own layer), pan the whole graph, and zoom-in or out of an area of interest. Additionally, the user is able to toggle on/off the display of each individual layer (with the exception of the initial list of nodes).

To avoid clutter, nodes linked in the PCI, MTI and TF layers that are connected to the same subset of initial genes are drawn as a single node, with only their cardinality shown. These nodes can be un-grouped by selecting the corresponding option from the *Node information* menu. All nodes in a layer can be grouped by selecting the icon next to the layer's name.

b. Enrichment graph

The *Enrichment graph* is included in the report page used for a list of genes. This graph has two distinctive areas, one dedicated to user interaction and the table display of the enrichment results (see Fig. 6); and a second one for the graph itself. Notice that enrichment result information is shown using two different types of display, a bar graph (shown in Fig. 7) or a heatmap plot (shown in Fig 8).

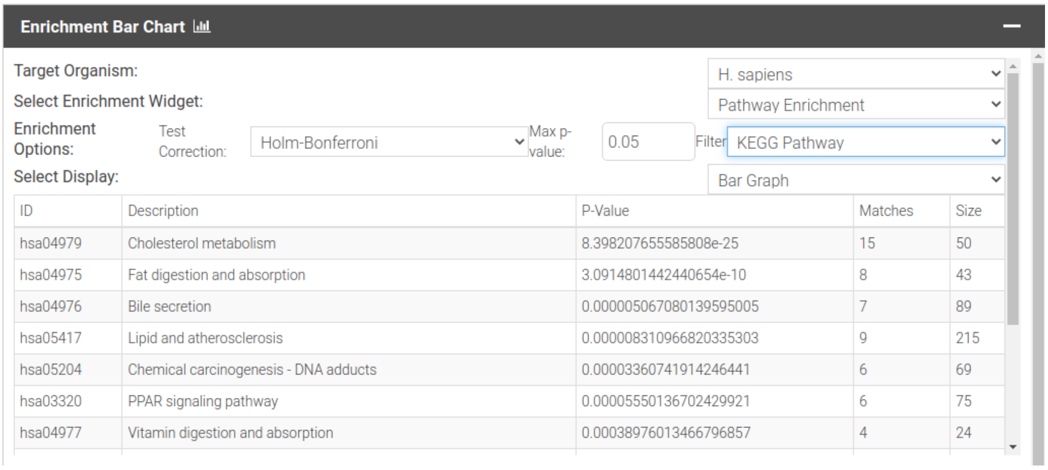

ID	Description	P-Value	Matches	Size
hsa04979	Cholesterol metabolism	8.398207655585808e-25	15	50
hsa04975	Fat digestion and absorption	3.0914801442440654e-10	8	43
hsa04976	Bile secretion	0.000005067080139595005	7	89
hsa05417	Lipid and atherosclerosis	0.000008310966820335303	9	215
hsa05204	Chemical carcinogenesis - DNA adducts	0.00003360741914246441	6	69
hsa03320	PPAR signaling pathway	0.00005550136702429921	6	75
hsa04977	Vitamin digestion and absorption	0.00038976013466796857	4	24

Figure 6: Enrichment graph User Interface and Results Table.

The user is allowed to modify the enrichment results through the following parameters:

- Target Organism - TargetMine includes Human, Mouse and Rat data, with this parameter allowing the user to select against which of these organisms to perform the enrichment.
- Enrichment Widget - select the type of enrichment to perform.
- Enrichment Options - select the correction test used for the enrichment results (None, Bonferroni, Holm-Bonferroni or Benjamini-Hochberg); the cut-off on the p-value used for display; and a filter, that depending on the selected widget, allows to choose the dataset on which enrichment results will be calculated.
- Display - choose between a bar graph or a heatmap display.

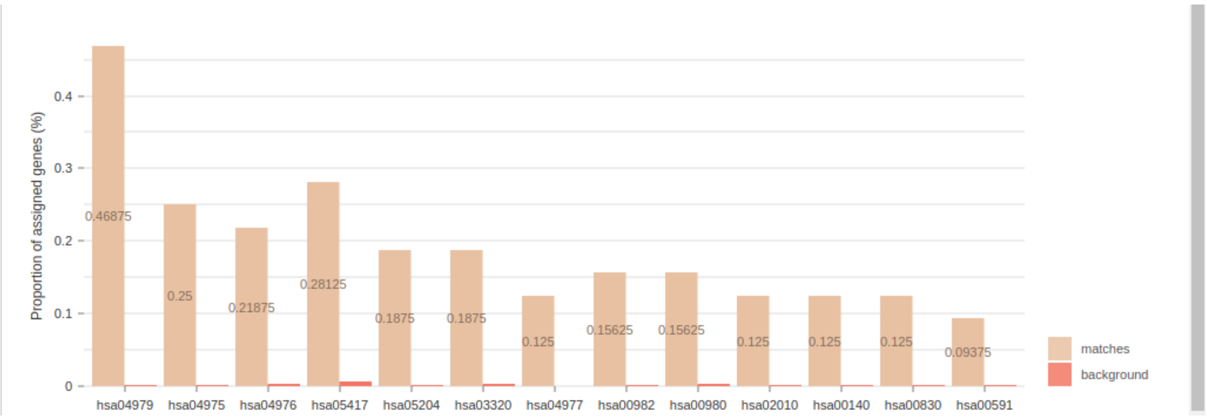

Figure 7: Enrichment graph. Bar graph display.

When a bar-chart display option is selected, the proportion of genes in the list with the corresponding annotation (for example, a given pathway) is shown as trace *matches*, whilst the proportion of genes in the entire genome with the same annotation is shown as the *background* trace.

On the other hand, when the heatmap display option is selected, a one-hot encoded version of the enriched pathways is shown. This representation highlights the genes in the original list that are actually a match for each enriched result. In the example shown in Figure 4, 15 genes in the list can be found in pathway *hsa04979 Cholesterol metabolism,* all of them clearly identifiable by looking at the first row of the matrix.

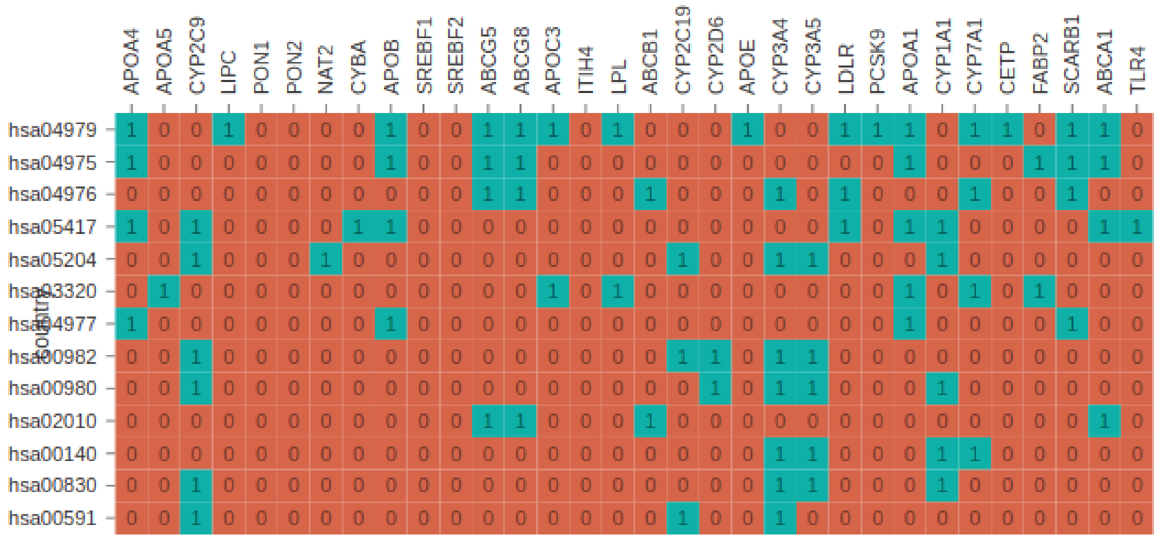

Figure 8: Enrichment graph. HeatMap display.

c. Gene Expression graph

A *Gene Expression Graph* is now included in the report page used for individual gene elements in TargetMine. The graph displays the HBI expression values stored at every probe associated with the target Gene stored in the system, using a linear scale. Values are grouped according to the tissue where the expression value was measured (see Fig. 9).

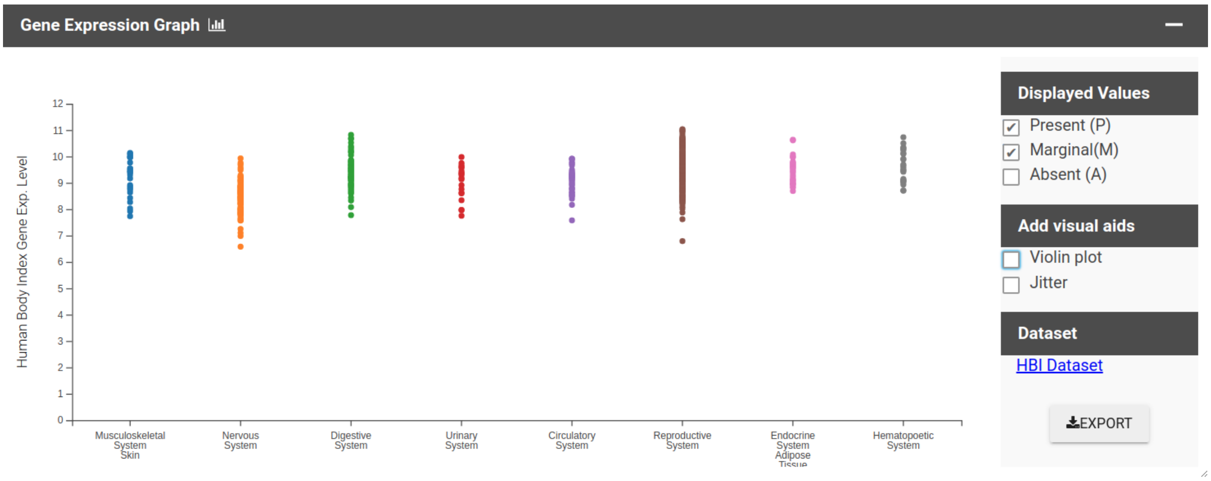

Figure 9: Sample Gene Expression Graph. Color is used to identify samples associated with different tissues.

Currently, there are two different ways in which users can interact with the data contained in the graph. First, by left or right clicking the labels of the graph, the user is able to expand and contract the levels of specificity at which the samples are shown. Currently there are three levels available. Fig. 10 shows an example of how inner categories of the Endocrine System / Adipose Tissue can be reached through two sequential expansion operations.

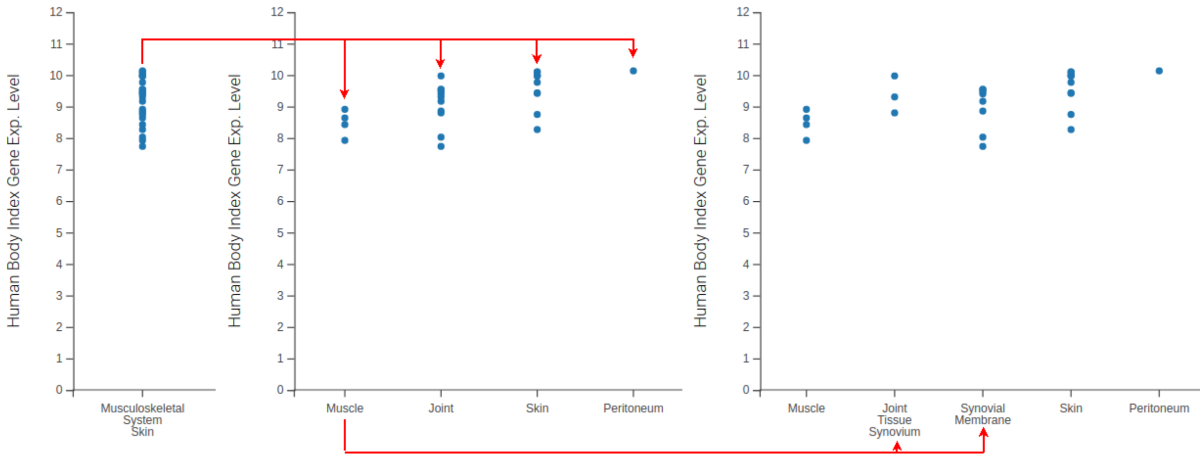

Figure 10: Expansion of Tissue levels. Notice that different levels might retain the same name, as is the case with "Adipose Tissue" that is kept on the second and third levels. Notice that right clicking (contracting) a level 2 label will also contract all level 3 labels of the same category into the highest level of sample tissue.

Second, the plot can also be modified with the inclusion of jitter and violin representations of the distribution of the samples. Both these additions can be selected and unselected through the checkboxes located in the top right corner of the graph. Notice that, once selected, the visual cue is independently applied to all currently displayed categories in the graph, including expanded levels of organization.

d. Bio-Activity graph

The *Bio-Activity graph* has been added to the report page of Chemical Compounds. It displays the activity concentration measured for the compound's target proteins, grouped per type, as shown in Fig. 11.

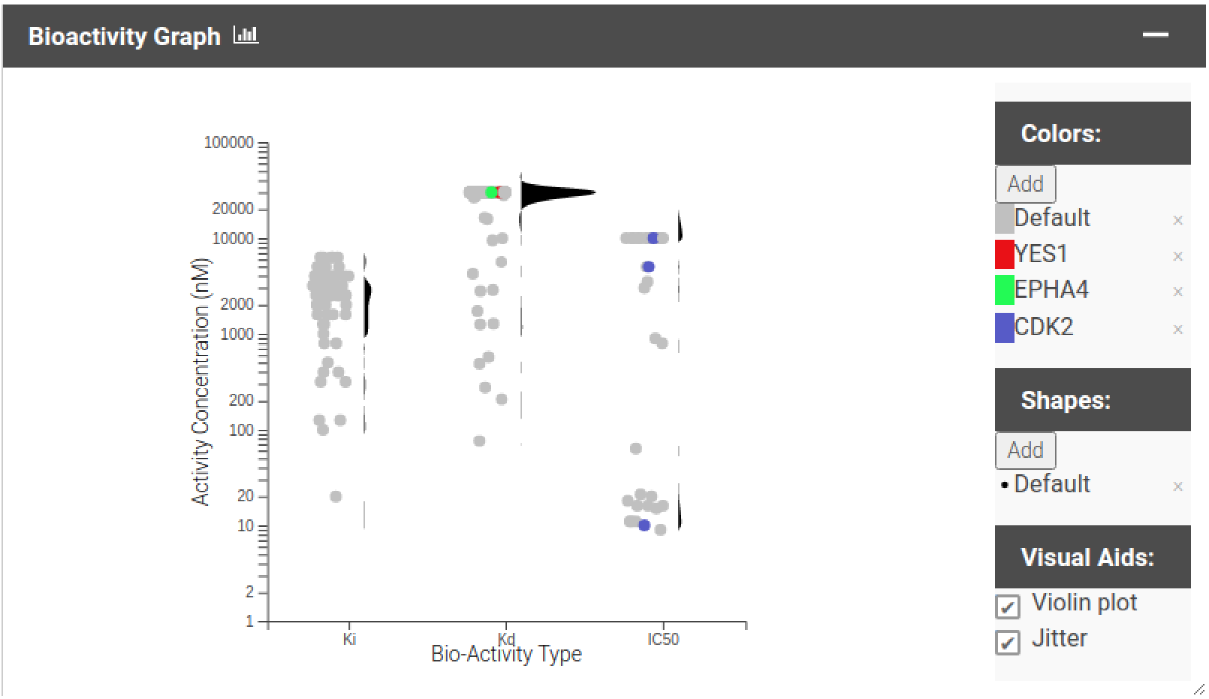

Figure 11: Bio-activity graph for palbociclib. Notice that violin plots and a jitter effect have been added to the graph, together with the use of color for 3 different genes.

As with the Gene Expression graph, the user is able to use violin plots and jitter to avoid occlusion of measurements. Additionally, it is also possible to change the color and/or shape of specific samples, as shown in Fig. 11 with the addition of color for the samples corresponding to three different targets.

To add a new color (or shape) simply click on the corresponding 'Add' button and choose from the available options in the pop-up menu. By clicking the small cross next to a color/shape, the corresponding display is removed from the list, and all the matching samples are displayed using the default parameters.

3. Bluegenes Migration

When migrating towards the use of the new Bluegenes Front-End, several software elements that had been previously coded for the Java-Server Page version of TargetMine needed to be refactored into *BlueGenes tools*. The following list provides an extensive recount of them.

Notice that, as effectively each individual widget that is added to any report page is considered now its own project, they can be individually stored and maintained. In consequence, for each element, a corresponding software repository is also specified[1].

[1] To avoid an excessive use of long links, only the name of the repository is indicated. All repositories can be found in TargetMine's organizational account on Github: https://github.com/targetmine/.

a. Drug Classification Hieararchy (bluegenes-drug-classification) - displays the hierarchy of the Anatomical Therapeutic Chemical Classification, and the Japan Standard Commodity Classification.

b. Alternative Forms (bluegenes-chembl-alternative-forms) - displays alternate forms of compounds in ChEMBL within TargetMine.

c. Compound Structure (bluegenes-compound-structure-image) - retrieves compound structure images for compounds either from the ChEMBL web service or the CACTUS web server.

d. Gene Expression Barcode (bluegenes-barcode-summary) - summarizes the Gene Expression Barcode 3.0 data integrated in TargetMine for the Genes.

e. Protein Annotation Summary (bluegenes-protein-annotation) - summarizes protein annotations in tables, including UniProt comments, UniProt features, and protein modifications.

f. Cytoscape Interaction Network (bluegenes-tm-cytoscape) - combines the functionalities of intermine's cytoscape-intermine and bluegenes-tool-cytoscape projects into a single tool for the display of interacting PPIs.

g. Disease Summary (bluegenes-gene-disease-pair) - provides a summary of disease related annotations that include the inferred genetic diseases form GWAS Catalog, ClinVar and SNP related publications in dbSNP, and disease annotations from other databases (like DisGeNET).

h. Gene Ontology Annotations (bluegenes-gene-ontology) - display gene ontology annotations (biological process, molecular function and cellular component) for Genes and Proteins.

i. Homology Info Viewer (bluegenes-homology) - summarizes protein orthologs compiled by TargetMine with homology data from other sources (including KEGG Orthology and HomoloGene) and orthologs from the annotation pipeline in NCBI.

j. Protein Domain Graph (bluegenes-tool-proteindomaingraph) - displays a graph that summarizes different domain annotations associated with an individual protein. Annotation information includes Domain, Homologous superfamily, Family and Conserved sites.
